# Excessive daytime sleepiness in nursing technicians: association with sleep quality and memory

**DOI:** 10.1590/0034-7167-2023-0332

**Published:** 2024-07-29

**Authors:** Renato Canevari Dutra da Silva, Ana Clara Barros Ribeiro, Maryanna Freitas Alves, Gilson Gonçalves Silva, Elton Brás Camargo

**Affiliations:** I Universidade de Rio Verde. Rio Verde, Goiás, Brazil

**Keywords:** Disorders of Excessive Somnolence, Sleep Quality, Memory, Nursing, Nursing, Team, Trastornos de Somnolencia Excesiva, Calidad del sueño, Memoria, Enfermería, Grupo de Enfermería

## Abstract

**Objective:**

to investigate excessive daytime sleepiness prevalence among nursing technicians and the association with sleep quality and memory.

**Methods:**

a cross-sectional, inferential study, carried out in a hospital unit in the state of Goiás between December 2020 and January 2021. Assessments were carried out using the Epworth Sleepiness Scale, the Pittsburgh Sleep Quality Index and the Prospective and Retrospective Memory Questionnaire, instruments validated for the Brazilian context. Bivariate and multivariate logistic regression analyzes were performed.

**Results:**

the sample consisted of 189 nursing technicians with a 40.9% excessive daytime sleepiness prevalence. In multivariate models, excessive daytime sleepiness was not associated with sleep quality, however there was a significant association with overall memory failures.

**Conclusions:**

study results demonstrate a high excessive daytime sleepiness occurrence, an association with overall memory failures and the need for psychosocial interventions for nursing technicians.

## RESUMEN

**Objetivo:**

investigar la prevalencia de somnolencia diurna excesiva entre técnicos de enfermería y la asociación con la calidad del sueño y la memoria.

**Métodos:**

estudio transversal, inferencial, realizado en una unidad hospitalaria del estado de Goiás entre diciembre de 2020 y enero de 2021. Las evaluaciones se realizaron mediante la Epworth Sleepiness Scale, Pittsburgh Sleep Quality Index y Prospective and Retrospective Memory Questionnaire, instrumentos validados para el contexto brasileño. Se realizaron análisis de regresión logística bivariados y multivariados.

**Resultados:**

la muestra estuvo compuesta por 189 técnicos de enfermería con una prevalencia de 40,9% de somnolencia diurna excesiva. En modelos multivariados, la somnolencia diurna excesiva no se asoció con la calidad del sueño, sin embargo, hubo una asociación significativa con fallas generales de memoria.

**Conclusiones:**

los resultados del estudio demuestran una alta ocurrencia de somnolencia diurna excesiva, asociación con fallas de memoria general y la necesidad de intervenciones psicosociales para los técnicos de enfermería.

## INTRODUCTION

Excessive daytime sleepiness (EDS) is a public health concern characterized as a relevant clinical symptom related to the difficulty in remaining awake and alert during the main episodes of wakefulness, with sleep occurring unintentionally or at inappropriate times during the waking period^(1)^. This condition can be a result of a variety of behaviors that lead to insufficient or fragmented sleep as well as being related to the consequences of underlying sleep disturbance^(2)^. The identification of factors associated with EDS is, therefore, of significant relevance to contribute to promotion of individuals’ occupational health.

EDS is often associated with negative consequences in the professional context, due to its influence on reduced cognitive function, delayed reaction time, feelings of fatigue and difficulty in logical reasoning^(3)^. This variety of effects can represent a considerable problem, especially for healthcare professionals, with the potential to negatively affect performance and patient care^(4)^. Specifically in hospital settings, nursing technicians represent one of the largest contingents of professionals playing a crucial role in patient care. It is also important to mention that nursing technicians in hospital units often organize their work activities into 12-hour shifts, which are distributed between day and night shifts. This practice, in itself, may present a potential contribution to the development of EDS^(5)^. EDS is associated with these professionals’ quality of sleep, and the association between low levels of sleep quality and EDS may have direct implications for work safety and efficiency^(6)^.

Furthermore, scientific literature has shown, through previously published research, that EDS may also be associated with cognitive declines, including, for instance, memory failure occurrence^(7-8)^. In this regard, it is important to highlight that EDS can compromise the retention of critical information for patient safety, especially among healthcare professionals responsible for direct assistance, such as nursing technicians, due to continuous contact with patients^(9)^.

Therefore, it is important to mention that the reported studies assess different categories of healthcare professionals^(9-10)^, and most of them were conducted in contexts other than Brazilian ones^(4,6,11)^. Furthermore, there are few studies that have assessed the specific association between EDS, sleep quality and memory failures in nursing technicians.

## OBJECTIVE

To investigate EDS prevalence and the association with sleep quality and memory among nursing technicians.

## METHODS

### Ethical aspects

The study received approval from the Research Ethics Committee of the proposing institution, and was conducted following the ethical precepts established in Resolutions 466/2012 and 588/2018. All participants provided their voluntary and informed consent by signing the Informed Consent Form.

### Study design, period and place

The study was conducted using a cross-sectional, analytical design with a quantitative approach. To ensure quality and transparency in presentation of results, the present study adopted STRengthening the Reporting of OBservational studies in Epidemiology (STROBE) as a methodological tool. Data collection was carried out between December 2020 and January 2021 in a medium-sized public hospital unit located in Rio Verde, in the state of Goiás.

### Population or sample; inclusion and exclusion criteria

The sample was recruited using a convenience sampling technique, and the sampling power was therefore calculated. Taking into account a sample of 198 recruited nursing technicians, a significance level of 0.05 and a medium effect size, the sampling power is approximately 0.85. Therefore, there is about an 85% probability that the results detected a real effect in data analysis^(12)^.

Nursing technicians over 18 years old, working in direct patient care and with at least six months of employment, with the aim of reducing selection bias and ensuring that professionals have more consistent work experience and representative of the target population, were included. Two professionals who reported having a diagnosis of sleep disturbance were excluded.

### Study protocol

Data collection was carried out through in-person interviews with professionals duly trained to apply the instruments. The interviews were carried out in a room reserved for this purpose, located in participants’ work environment. In order to guarantee professional participation, after their working day, they were approached and informed about the objectives of this research. After consent to participate, the interview was scheduled according to the availability of each participant. The interviews lasted an average of 25 minutes, and data were collected using a validated instrument.

EDS assessment was performed using the Epworth Sleepiness Scale (ESS). The ESS is a self-reported questionnaire, validated for use in Brazil^(12)^, which measures the probability of falling asleep or dozing in eight daily situations. Each situation is assessed on a scale of 0 to 3, where 0 represents “no chance of dozing” and 3 represents “high chance of dozing”. The total score ranges from 0 to 24, with higher scores indicating greater daytime sleepiness. In the present research, the cut-off point ≥10 was used to define the presence of EDS among nursing technicians, as described by the instrument author^(13)^. The instrument’s internal consistency presented a Cronbach’s alpha value of 0.87 in the present study.

The instrument used to assess sleep quality was the Pittsburgh Sleep Quality Index (PSQI). The PSQI is a questionnaire that assesses an individual’s sleep quality and patterns over the past four weeks. It consists of 19 items, distributed into seven main components, such as overall sleep quality, sleep latency, sleep duration, sleep efficiency, sleep disturbance, use of sleeping medication and daytime dysfunction due to sleepiness^(14)^. Each component is scored on a scale of 0 to 3, and the total PSQI score can range from 0 to 21 points. In the present study, professionals were categorized as having good sleep quality (PSQI ≤ 5) or poor sleep quality (PSQI > 5), according to the validity study for the Brazilian context^(15)^. The instrument’s internal consistency, in the present study, presented a Cronbach’s alpha index of 0.78.

Memory assessment was carried out using the Prospective and Retrospective Memory Questionnaire (PRMQ), validated for Brazil^(16)^. The PRMQ is a questionnaire composed of 16 items that aims to assess everyday memory failures, focusing on two specific areas of memory: prospective memory and retrospective memory. Furthermore, the instrument also allows assessing a overall memory factor. The results are obtained by adding the items together, resulting in an overall score of 16 to 80 points. For prospective and retrospective memory subscales, the maximum possible scores are 40 points each. In the present study, a cut-off point of 20 points was used, indicating the presence of significant failures in overall memory. Participants were then categorized as having absence or presence of overall, prospective and retrospective memory failures^(17)^. The total PRMQ scale reliability, as well as prospective and retrospective subscales, presented Cronbach’s alpha values with coefficients of 0.86, 0.85 and 0.82, respectively.

The covariables used in the study included sociodemographic characteristics, including, for instance, sex, age and skin color. Clinical covariables included variables related to participants’ physical and mental health, such as Body Mass Index (BMI), anxiety and depression report, in addition to self-perceived health. Behavioral assessments included alcohol consumption at least once during the week, tobacco, ultra-processed foods, among others. The work variables included characteristics related to nursing technicians’ work environment, such as job tenure and daily workload.

### Analysis of results, and statistics

To build the database, the procedure of double typing responses was adopted in Excel for Windows^®^, later exported to Statistical Package for the Social Sciences^®^.

Descriptive statistical analyzes were carried out to characterize the sample and variables of interest in the study, and are presented using absolute and relative frequencies. Bivariate and multivariate logistic regression analyzes were used to investigate factors associated with EDS. Initially, bivariate logistic regression analyzes were performed, in which each independent variable was tested individually in relation to the presence of EDS. The variables that presented a p-value < 0.2 were selected for inclusion in the multivariate model.

Soon thereafter, multivariate logistic regression analysis was performed using the forward method, using a model that included step by step all the independent variables selected in the previous step (p < 0.2). This analysis made it possible to evaluate the joint effect of independent variables on the presence of EDS, controlling possible confounding factors. Odds Ratios (OR) were estimated with 95% Confidence Intervals (95%CI) for each independent variable included in the multivariate model. A p-value < 0.05 was considered as a criterion for statistical significance to assess the association between independent variables and the presence of EDS in the multivariate model. The multivariate logistic regression assumptions were assessed by variance inflation values and tolerance statistic, which presented values below 1.35 and 0.93, respectively. Statistical analyzes were performed using the Statistical Package for the Social Sciences^®^ version 24, with a license acquired by the study authors themselves.

## RESULTS

The study was carried out with 198 nursing technicians, the majority of whom were female (95.5%) and with a mean age of 37.2 years. Most participants were black (81.8%), did not have a partner (60.6%) and had less than 15 years of education (68.2%). Regarding professional experience, 57.6% of participants had up to six years of experience. Most participants reported working up to 12 hours a day (66.7%). Most participants (93.9%) classified their sleep quality as poor, and 43.9% reported sleep deprivation, with less than six hours of sleep. As for memory, 37.9% of participants reported overall memory failures, 10.6%, prospective memory failures, and 3%, retrospective memory failures. Furthermore, Table 1 shows the sample characteristics.

**Table 1:** Sociodemographic, work, behavioral, clinical characteristics, sleep quality and memory failures of nursing technicians. Rio Verde, Goiás, Brazil, 2020-2021

Variables	N	%
Sex		
Female	189	95.5
Male	9	4.5
Mean age (standard deviation)	37,2	± 8.1
Skin color		
White	36	18.2
Black	162	81.8
Marital status		
No partner	78	39.4
With partner	120	60.6
Education		
Less than 15 years	135	68.2
More than 15 years	63	31.8
Job tenure		
≤ six years	114	57.6
> six years	84	42.4
Working hours/day		
≤ 12 hours	132	66.7
> 12 hours	66	33.3
Fruit and vegetable consumption		
Inadequate	81	40.9
Adequate	117	59.1
Ultraprocessed food consumption		
Does not consume	63	31.8
Consumes	135	68.2
Alcohol consumption		
No	60	30.3
Yes	138	69.7
Coffee consumption		
No	33	16.7
Yes	165	83.3
Tobacco use		
No	174	87.9
Yes	24	12.1
Body Mass Index		
Normal weight/underweight	78	39.4
Overweight	63	31.8
Obesity	57	28.8
Depression		
No	174	87.9
Yes	24	12.1
Anxiety		
No	114	57.5
Yes	84	42.4
Self-perceived health		
Good/very good	141	71.2
Poor/very poor	57	28.8
Sleep quality		
Good quality	12	6.1
Poor quality	186	93.9
Sleep deprivation		
No deprivation/six hours or more	111	56.1
Deprivation/less than six hours	87	43.9
Overall memory failures		
Absent	123	62.1
Present	75	37.9
Prospective memory failures		
Absent	177	89.4
Present	21	10.6
Retrospective memory failures		
Absent	192	97
Present	6	3

When carrying out EDS assessment in the sample of nursing technicians, it was found that 81 (40.9%, 95%CI: 33.8 - 47.5) of these professionals were classified as having EDS and 117 (59.7 %, 95%CI: 52.5 - 66.2) were classified as absence of EDS.

The results of bivariate logistic regression demonstrate associations between several independent variables and EDS. Professionals with partners, with less education, who consumed ultraprocessed foods and alcohol, were more likely to have EDS. BMI was also a relevant factor, with overweight and obese people having a greater chance of having EDS compared to those with normal weight or underweight. Self-reported depression, anxiety, poor/very poor perception of health and overall memory failures were also associated with EDS (Table 2).

**Table 2:** Frequency of excessive daytime sleepiness and association with sociodemographic, work, behavioral, clinical variables, sleep quality and memory failures among nursing technicians. Rio Verde, Goiás, Brazil, 2020-2021

Variables	Presence of excessive daytime sleepiness n (%) 81 (40.9)	OR (95%CI)^*^	*p*
Sex			
Female	75 (92.5)	0.32 (0.07 - 1.36)	0.124
Male	6 (7.4)	Reference	
Skin color			
White	15 (18.51)	Reference	
Black	66 (81.48)	0.96 (0.46 - 2)	0.919
Marital status			
No partner	24 (29.63)	Reference	
With partner	57 (70.37)	2.03 (1.11 - 3.7)	0.020
Education			
Less than 15 years	63 (77.77)	2.18 (1.15 - 4.16)	0.017
More than 15 years	18 (22.22)	Reference	
Job tenure			
≤ six years	54 (66.66)	Reference	
> six years	27 (33.33)	0.52 (0.29 - 0.94)	0.032
Working hours/day			
≤ 12 hours	51 (62.96)	Reference	
> 12 hours	30 (37.03)	1.32 (0.72 - 2.40)	0.358
Fruit and vegetable consumption			
Inadequate	36 (44.44)	1.28 (0.72 - 2.27)	0.400
Adequate	45 (55.55)	Reference	
Ultraprocessed food consumption			
Does not consume	18 (22.22)	Reference	
Consumes	63 (77.77)	2.18 (1.15 - 4.16)	0.017
Alcohol consumption			
No	12 (14.81)	Reference	
Yes	69 (85.18)	4 (1.95 - 8.17)	<0.001
Coffee consumption			
No	15 (18.51)	Reference	
Yes	66 (81.48)	0.80 (0.37 - 1.70)	0.561
Tobacco use			
No	69 (85.18)	Reference	
Yes	12 (14.81)	1.52 (0.64 - 3.58)	0.336
Body Mass Index			
Normal weight/underweight	21 (25.92)	Reference	
Overweight	33 (40.74)	2.98 (1.47 - 6.03)	0.002
Obesity	27 (33.33)	2.44 (1.18 - 5.02)	0.015
Depression			
No	63 (77.77)	Reference	
Yes	18 (22.22)	5.28 (1.99 - 14)	<0.001
Anxiety			
No	39 (48.14)	Reference	
Yes	42 (51.85)	1.92 (1.08 - 3.42)	0.026
Self-perceived health			
Good/very good	42 (51.85)	Reference	
Poor/very poor	39 (48.14)	5.10 (2.62 - 9.93)	<0.001
Sleep quality			
Good quality	3 (3.70)	Reference	
Poor quality	78 (96.29)	2.16 (0.56 - 8.26)	0.258
Sleep deprivation			
No deprivation/six hours or more	45 (55.55)	Reference	
Deprivation/less than six hours	36 (44.44)	1.03 (0.58 - 1.83)	0.905
Overall memory failures			
Absent	33 (40.74)	Reference	
Present	48 (59.26)	4.84 (2.61 - 8.99)	<0.001
Prospective memory failures			
Absent	69 (85.18)	Reference	
Present	14 (14.81)	2.08 (0.93 - 5.21)	0.115
Retrospective memory failures			
Absent	75 (92.59)	Reference	
Present	6 (7.40)	2.44 (0 - 0.01)	0.986

* *Odds Ratio (95% Confidence Interval).*

In the multivariate logistic regression analysis, the variables that remained associated with EDS are found in the table. In relation to sex, women were 95% less likely to have EDS compared to men (p = 0.001). Furthermore, job tenure was also a protective factor, in which professionals with more than six years of career had a 64% lower chance of having EDS compared to those with less experience (p = 0.020). Alcohol consumption showed a significant association with EDS, with a 5.71 times greater chance for alcohol consumers compared to non-consumers (p = 0.002). Self-reported depression also showed a significant association with the outcome, with a 3.66 times greater chance for those with depression compared to those without depression (p = 0.028). Self-perceived health poor/very poor showed a significant association with the outcome, with a 15.41 times greater chance compared to those who reported a good/very good health perception (p < 0.001). Regarding overall memory failure, individuals with memory failures were 3.32 times more likely to have EDS compared to those who did not have memory failures (p = 0.002) (Table 3).

**Table 3:** Association of variables with excessive daytime sleepiness among nursing technicians in multivariate logistic regression analysis. Rio Verde, Goiás, Brazil, 2020-2021

Variables	aOR (95%CI)^*^	*p*
Sex		
Female	0.05 (0 - 0.29)	0.001
Male	Reference	
Job tenure		
≤ six years	Reference	
> six years	0.36 (0.15 - 0.85)	0.020
Alcohol consumption		
No	Reference	
Yes	5.71 (1.90 - 17.16)	0.002
Depression		
No	Reference	
Yes	3.66 (1.15 - 11.66)	0.028
Self-perceived health		
Good/very good	Reference	
Poor/very poor	15.41 (5.37 - 44.27)	<0.001
Overall memory failures		
Absent	Reference	
Present	3.32 (1.52 - 7.25)	0.002

* *aOR = adjusted Odds Ratio (95% Confidence Interval).*

## DISCUSSION

The results of this study reveal a high EDS prevalence among nursing technicians along with sociodemographic, work, behavioral and clinical factors associated with this condition. Furthermore, there was a significant association between EDS and overall memory failures, whereas no association was observed with sleep quality.

Of the nursing technicians assessed, 81 (40.9%) had EDS. This prevalence is higher than the results found in studies carried out with the general population, which also used the same assessment instrument. These studies reported a EDS prevalence ranging between 8.5% and 33%^(2,18)^. However, in the Brazilian context, research carried out with university students in the health field identified an EDS prevalence of 54.4%^(19)^, higher than that found in the present study. These results demonstrate the variability of EDS prevalence according to the population assessed, indicating the importance of specifically assessing the occurrence of this problem among nursing technicians.

In relation to nursing professionals, studies carried out in different international settings demonstrated a EDS prevalence of 25.6% among nurses in Norway^(20)^, 28% among nurses in England^(21)^ and 36.1% among nurses in South Korea^(22)^. In studies carried out with nursing professionals in Brazil, a EDS prevalence was observed varying between 30.09%^(23)^ and 38.6%^(9)^, values lower than those found in the present study. Among the possible reasons for the difference in the results found, they may refer to the moment in which the assessments were carried out, considering that, in the present study, collection was carried out during the COVID-19 pandemic. No studies carried out in a national context that assessed EDS in nursing professionals during the COVID-19 pandemic were identified. The COVID-19 pandemic influenced EDS among nursing technicians due to several factors, such as increased psychological stress, increased exposure to the virus, workload and changes in sleeping and life habits^(22,24)^. Hence, there is a need for longitudinal monitoring of these professionals with the aim of assessing the impact resulting from exposure to factors arising from the pandemic on nursing technicians’ physical and mental health.

Furthermore, it is important to mention that the relationship between EDS and nursing practice can be complex and multifaceted. The nature of nursing work, which often involves long working hours, night shifts, fast pace and intense physical and emotional demands, can contribute to EDS occurrence^(25)^.

Although no significant association with EDS was observed, it is important to highlight that the significant majority of the sample of nursing technicians had poor sleep quality, representing a total of 186 individuals (93.9%). These results indicate that the population studied is susceptible to sleep quality-related problems^(26)^, even if they are not directly associated with EDS occurrence. Recent research involving 42 hospital nurses in southern Brazil revealed that 64.3% of professionals had poor sleep quality^(27)^. Furthermore, another study conducted with nursing professionals identified a prevalence of 75% of individuals assessed with sleep disturbance^(25)^. These findings support the results found in the present study, highlighting the worrying situation of sleep quality among nursing professionals nationally. The high proportion of nursing professionals with poor sleep quality may be a reflection of challenging working conditions and the impact of intense and irregular work routines on adequate rest^(28-29)^.

Among the covariables that showed a significant association with EDS, women and professionals with longer experience were less likely to present this problem. Women are less likely to have EDS due to regular patterns and better sleep quality compared to men^(30)^. Furthermore, professional experience may be associated with EDS, possibly due to the development of sleep management strategies over the years, mitigating the negative impacts^(31)^.

The other covariates associated with EDS among nursing technicians were alcohol consumption, self-reported depression and negative self-perceived health. In relation to alcohol, despite having sedative and hypnotic properties, its consumption can lead to fragmentation, alteration and reduction of sleep quality, which increases the chances of developing EDS. There is evidence that alcohol consumption can interfere with sleep processes, negatively affecting its structure and duration^(32)^. In the context of depression, the results are in agreement with data found in a study carried out in China with nurses from a general hospital, which demonstrated depression as a predictor of EDS occurrence^(11)^. It is important to mention that EDS and depression can have a bidirectional relationship, in which each one influences the other, and this fact has been confirmed in studies that point out the effects of sleep problems on depression and vice versa^(33)^. Furthermore, the association of negative self-perceived health with EDS may be indicative of the consequences of poor sleep quality and a high rate of EDS, factors that result in cognitive deficits and mood changes, which can lead to a more negative health assessment.

The results of this study demonstrate a significant association between EDS and overall memory failures, even after controlling for possible confounding factors. However, no associations were found between EDS and prospective and retrospective memory. The association found is in line with the scientific literature on sleep, which has extensively examined the relationship between sleep and the efficiency of executive functions, including memory performance. A recent systematic review highlighted the influence of sleep aspects on memory functioning, further reinforcing the importance of these findings^(8)^. Supporting the results of this research, a study carried out with older adults in Japan showed EDS as an important predictor of subjective memory impairment^(34)^.

A possible explanation for the association between EDS and memory failures is related to attention and concentration deficits resulting from EDS. Individuals who have EDS tend to have difficulty maintaining focus and sustaining attention throughout the day, which can negatively affect memory performance^(1)^. When they are excessively sleepy during the day, they may encounter difficulties in properly encoding information as well as effectively retrieving it during memory tasks. These attention and concentration deficits can result in failures in acquisition, retention and retrieval of information stored in memory, thus compromising memory-related cognitive functioning^(35)^.

It is important to mention that EDS associated with memory failures, among nursing technicians, causes significant impacts on quality of life, professional performance and patient safety. This problem can affect the ability to concentrate, decision-making, motor coordination and vigilance, which can lead to errors and accidents in the workplace^(11)^. The results of a study carried out with nurses from a hospital unit in China demonstrated that EDS was associated with a 1.83-fold increase in adverse event occurrence^(11)^. These findings support the theory that EDS among nursing professionals represents a significant risk to patient safety due to the increased likelihood of adverse events during professional practice^(36)^.

Due to the high rates of EDS evidenced in the present study, it is worth highlighting the need for continuous assessment of aspects related to sleep among nursing technicians during the post-pandemic period. Therefore, it is essential to recognize that EDS-related problem may persist or evolve in subsequent events, requiring special attention and preventive strategies to mitigate its impacts on nursing technicians’ health, sleep and professional performance.

### Study limitations

The present study must be interpreted considering some relevant limitations that may impact the results and their generalization. The sample used in this study was restricted to a single healthcare institution, which may limit the representativeness of the results. Using self-report questionnaires may result in recall bias and may influence the accuracy of observed associations. Other study limitations refer to the fact that a detailed assessment was not carried out with a validated instrument on alcohol consumption and also related to the omnipresent presence of bias, since cognitive processes, social desirability and specific conditions can influence participants’ responses.

Therefore, future studies are needed to consider these limitations. New research with more representative samples, longitudinal designs and the use of more robust instruments to assess sleep quality, EDS and memory are necessary to provide more robust evidence and clarify the relationship between these variables.

### Contributions to nursing, health or public policy

The results of this research have important practical implications. The high EDS prevalence and the association with memory failures highlight the need for healthcare institutions and managers to provide education and awareness to nursing professionals about the risks associated with EDS for both the health of professionals and patients assisted. It is essential to include training aimed at developing strategies to improve sleep quality among nursing technicians. A promising approach in this regard is mindfulness practice, which consists of low-density exercises that can be easily incorporated into these professionals’ routine^(37-38)^. Studies have shown that regular mindfulness practice can improve sleep quality, reduce daytime sleepiness and promote overall well-being. Healthcare institutions must carry out EDS screening and assessment actions among nursing professionals and provide support and promote a culture of safety to encourage professionals to report EDS-related problems through open communication and mutual support.

The study findings, which identified 40.9% of EDS and an association with memory failures among nursing technicians, demonstrate that EDS is a public health concern that can affect healthcare professionals’ professional performance and quality of life. These results suggest the need to provide support for the category of healthcare professionals and, mainly, nursing, especially in post-pandemic periods. This support must cover psychosocial interventions, in addition to well-being programs and strategies to mitigate potential impacts of professional practice on these professionals’ mental health. Furthermore, it is recommended that future research assess detailed aspects related to professional practice, exploring psychosocial interventions aimed at reducing EDS and using effective approaches targeting memory failures associated with EDS.

## CONCLUSIONS

The results of this study demonstrate a high EDS occurrence and the association with overall memory failures among nursing technicians in multivariate models.

The high proportion of nursing technicians with EDS and the association with memory failures reinforce the need for interventions and health policies aimed at promoting adequate and healthy sleep in this population, aiming to improve their quality of life and performance in the work environment.
