# Supplementary material for: Excessive daytime sleepiness in nursing technicians: association with sleep quality and memory
Source: Rev Bras Enferm. 2024 Jul 29;77(3):e20230332. doi: 10.1590/0034-7167-2023-0332 (PMC11290731; doi:10.1590/0034-7167-2023-0332)
Supplement: Translation

# Translation: Sonolência diurna excessiva em técnicos de enfermagem: associação com qualidade do sono e memória

**DOI:** 10.1590/0034-7167-2023-0332pt

**Authors:** Renato Canevari Dutra da Silva, Ana Clara Barros Ribeiro, Maryanna Freitas Alves, Gilson Gonçalves Silva, Elton Brás Camargo

**Affiliations:** I Universidade de Rio Verde. Rio Verde, Goiás, Brasil

**Keywords:** Distúrbios do Sono por Sonolência Excessiva, Qualidade do Sono, Memória, Enfermagem, Equipe de Enfermagem

## Abstract

**Objetivo:**

investigar a prevalência de sonolência diurna excessiva entre técnicos de enfermagem e a associação com a qualidade do sono e memória.

**Métodos:**

estudo transversal, inferencial, realizado em uma unidade hospitalar no estado de Goiás entre dezembro de 2020 e janeiro de 2021. As avaliações foram realizadas por meio da *Epworth Sleepiness Scale*, do *Pittsburgh Sleep Quality Index* e da *Prospective and Retrospective Memory Questionnaire*, instrumentos validados para o contexto brasileiro. Foram realizadas análises de regressão logística bivariada e multivariada.

**Resultados:**

a amostra composta por 189 técnicos de enfermagem com prevalência de 40,9% de sonolência diurna excessiva. Em modelos multivariados, a sonolência diurna excessiva não associou com a qualidade do sono, no entanto houve associação significativa com falhas de memória geral.

**Conclusões:**

os resultados do estudo demonstram elevada ocorrência de sonolência diurna excessiva, associação com falhas de memória geral e a necessidade de intervenções psicossociais para técnicos de enfermagem.

## INTRODUÇÃO

A sonolência diurna excessiva (SDE) é um problema de saúde pública caracterizada como um sintoma clínico relevante relacionado à dificuldade em manter-se acordado e alerta durante os principais episódios de vigília, ocorrendo o sono de forma não intencional ou em horários inadequados do período de vigília^(1)^. Essa condição pode ser resultante de diversos comportamentos que conduzem a um sono insuficiente ou fragmentado, como também pode estar relacionado a consequências de distúrbios do sono subjacentes^(2)^. A identificação dos fatores associados a SDE é, portanto, de significativa relevância para contribuir com a promoção da saúde ocupacional dos indivíduos.

A SDE está frequentemente associada a consequências negativas no contexto profissional, devido à influência na redução da função cognitiva, no retardo do tempo de reação, na sensação de fadiga e na dificuldade de raciocínio lógico^(3)^. Essa variedade de efeitos pode representar um problema considerável, especialmente para os profissionais atuantes na área da saúde, tendo o potencial de afetar negativamente o desempenho e o atendimento ao paciente^(4)^. Especificamente no ambiente hospitalar, os técnicos de enfermagem representam um dos maiores contingentes de profissionais desempenhando um papel crucial na assistência aos pacientes. Também é importante mencionar que os técnicos de enfermagem nas unidades hospitalares frequentemente adotam uma organização das atividades laborais em turnos de 12 horas, os quais são distribuídos entre os turnos diurno e noturno. Essa prática, por si só, pode apresentar uma potencial contribuição para o desenvolvimento da SDE^(5)^. A SDE está associada à qualidade do sono desses profissionais, e a associação entre os baixos níveis de qualidade de sono e a SDE podem ter implicações diretas na segurança e eficiência do trabalho^(6)^.

Além disso, a literatura científica tem evidenciado, por meio de pesquisas publicadas anteriormente, que a SDE pode estar associada também com declínios cognitivos, incluindo, por exemplo, a ocorrência de falhas de memória^(7-8)^. Nesse sentido, é importante ressaltar que a SDE pode comprometer a retenção de informações críticas para a segurança do paciente, especialmente entre os profissionais de saúde responsáveis pela assistência direta, como é o caso dos técnicos de enfermagem, devido ao contato contínuo com os pacientes^(9)^.

Portanto, é relevante mencionar que os estudos relatados avaliam variadas categorias de profissionais de saúde^(9-10)^, e a maioria deles foi conduzida em contextos distintos do brasileiro^(4,6,11)^. Além disso, são escassos os estudos que avaliaram a associação específica entre a SDE, a qualidade do sono e as falhas de memória em técnicos de enfermagem.

## OBJETIVO

Investigar a prevalência de SDE e a associação com a qualidade do sono e memória entre técnicos de enfermagem.

## MÉTODOS

### Aspectos éticos

O estudo recebeu a aprovação do Comitê de Ética em Pesquisa da instituição proponente, e foi conduzido seguindo os preceitos éticos estabelecidos nas Resoluções n^o^ 466/2012 e n^o^ 588/2018. Todos os participantes forneceram seu consentimento voluntário e informado por meio da assinatura do Termo de Consentimento Livre e Esclarecido.

### Desenho, período e local do estudo

O estudo foi conduzido por meio de um desenho transversal, analítico e com abordagem quantitativa. Para assegurar a qualidade e a transparência na apresentação dos resultados, o presente estudo adotou o *STrengthening the Reporting of OBservational studies in Epidemiology* (STROBE) como uma ferramenta metodológica. A coleta de dados foi realizada entre os meses de dezembro de 2020 e janeiro de 2021 em uma unidade pública hospitalar de médio porte localizada em Rio Verde, no estado de Goiás.

### População ou amostra; critérios de inclusão e exclusão

A amostra foi recrutada por meio de técnica de amostragem por conveniência, sendo, portanto, realizado o cálculo do poder amostral. Levando em consideração uma amostra de 198 técnicos de enfermagem recrutados, um nível de significância de 0,05 e um tamanho de efeito médio, o poder amostral é de aproximadamente 0,85. Sendo assim, há cerca de 85% de probabilidade de os resultados terem detectado um efeito real nas análises de dados^(12)^.

Foram incluídos no estudo profissionais de enfermagem de nível técnico, maiores de 18 anos, atuantes na assistência direta ao paciente e com, no mínimo, seis meses de vínculo empregatício, com o objetivo de reduzir o viés de seleção e garantir que os profissionais tenham experiência de trabalho mais consistente e representativa da população-alvo. Foram excluídos dois profissionais que relataram apresentar diagnóstico de distúrbios do sono.

### Protocolo do estudo

A coleta de dados foi realizada por meio de entrevista face a face com profissionais devidamente treinados para aplicação dos instrumentos. As entrevistas foram realizadas em uma sala reservada para essa finalidade, localizada no ambiente de trabalho dos participantes. Com vistas a garantir a participação dos profissionais, após a jornada de trabalho, os mesmos foram abordados e informados sobre os objetivos da pesquisa. Após o consentimento da participação, a entrevista foi agendada de acordo com a disponibilidade de cada participante. As entrevistas duraram em média 25 minutos, e os dados foram coletados por meio de instrumento validados.

A avaliação da SDE foi realizada utilizando a Escala de Sonolência de Epworth (*Epworth Sleepiness Scale* - ESS). A ESS é um questionário autorrelatado, validado para uso no Brasil^(12)^, que mede a probabilidade de adormecer ou cochilar em oito situações do cotidiano. Cada situação é avaliada em uma escala de 0 a 3, em que 0 representa “nenhuma chance de cochilar” e 3 representa “muita chance de cochilar”. A pontuação total varia de 0 a 24, sendo que pontuações mais altas indicam maior sonolência diurna. Na presente pesquisa, utilizou-se o ponto de corte **≥**10 para definir a presença de SDE entre os técnicos de enfermagem, conforme descrito pelo autor do instrumento^(13)^. A consistência interna do instrumento apresentou valor de alfa de Cronbach de 0,87 no presente estudo.

O instrumento utilizado para avaliar a qualidade do sono foi o Índice de Qualidade do Sono de Pittsburgh (*Pittsburgh Sleep Quality Index* - PSQI). O PSQI é um questionário que avalia a qualidade e os padrões de sono de um indivíduo nas últimas quatro semanas. É composto por 19 itens, distribuídos em sete componentes principais, tais como qualidade subjetiva do sono, latência do sono, duração do sono, eficiência do sono, distúrbios do sono, uso de medicamentos para dormir e disfunção diurna^(14)^. Cada componente é pontuado em uma escala de 0 a 3, sendo que a pontuação total do PSQI pode variar de 0 a 21 pontos. No presente estudo, os profissionais foram categorizados com boa qualidade do sono (PSQI ≤ 5) ou má qualidade do sono (PSQI > 5), de acordo com o estudo de validação para o contexto brasileiro^(15)^. A consistência interna do instrumento, no presente estudo, apresentou índice de alfa de Cronbach de 0,78.

A avaliação da memória fora realizada pelo instrumento Questionário de Memória Prospectiva e Retrospectiva (*Prospective and Retrospective Memory Questionnaire* - PRMQ), validado para o Brasil^(16)^. O PRMQ é um questionário composto por 16 itens que tem como objetivo avaliar as falhas de memória cotidianas, com foco em duas áreas específicas da memória: a memória prospectiva e a memória retrospectiva. Além disso, o instrumento também permite a avaliação de um fator de geral de memória. Os resultados são obtidos por meio da soma dos itens, resultando em um escore global de 16 a 80 pontos. Para as subescalas da memória prospectiva e retrospectiva, os escores máximos possíveis são de 40 pontos cada. No presente estudo, foi utilizado um ponto de corte de 20 pontos, indicando a presença de falhas significativas na memória geral. Os participantes foram então categorizados como tendo ausência ou presença de falhas de memória geral, prospectiva e retrospectiva^(17)^. A confiabilidade da escala total do PRMQ, bem como das subescalas prospectiva e retrospectiva, apresentou valores de alfa de Cronbach com coeficientes de 0,86, 0,85 e 0,82, respectivamente.

As covariáveis utilizadas no estudo contemplaram características sociodemográficas, incluindo, por exemplo, sexo, idade e cor da pele. As covariáveis clínicas englobaram variáveis relacionadas à saúde física e mental dos participantes, como o Índice de Massa Corporal (IMC), relato de ansiedade e depressão, além da percepção de saúde. As avaliações comportamentais incluíram o consumo de álcool pelo menos uma ocasião durante a semana, tabaco, alimentos ultraprocessados, entre outros. As variáveis laborais contemplaram características relacionadas ao ambiente de trabalho dos técnicos de enfermagem, como o tempo de profissão e a carga horária diária.

### Análise dos resultados e estatística

Para a construção do banco de dados, adotou-se o procedimento de dupla digitação das respostas no *software Excel for Windows* ^®^, posteriormente exportado para o *software Statistical Package for the Social Sciences^®^.*

Análises estatísticas descritivas foram realizadas para caracterizar a amostra e as variáveis de interesse do estudo, e são apresentadas por meio de frequências absolutas e relativas. Foram utilizadas análises de regressão logística bivariada e multivariada para investigar os fatores associados à SDE. Inicialmente, foram realizadas análises de regressão logística bivariada, em que cada variável independente foi testada individualmente em relação à presença de SDE. As variáveis que apresentaram um valor de p < 0,2 foram selecionadas para inclusão no modelo multivariado.

Em seguida, foi realizada a análise de regressão logística multivariada, pelo método *forward*, utilizando um modelo que incluiu passo a passo todas as variáveis independentes selecionadas na etapa anterior (p < 0,2). Essa análise permitiu avaliar o efeito conjunto das variáveis independentes sobre a presença de SDE, controlando possíveis fatores de confusão. Foram estimados os *Odds Ratios* (OR) com Intervalos de Confiança de 95% (IC95%) para cada variável independente incluída no modelo multivariado. Foi considerado um valor de p < 0,05 como critério de significância estatística para avaliar a associação entre as variáveis independentes e a presença de SDE no modelo multivariado. Os pressupostos da regressão logística multivariada foram avaliados pelos valores de inflação da variância e pela estatística de *tolerance*, os quais apresentaram valores abaixo de 1,35 e 0,93, respectivamente. As análises estatísticas foram realizadas pelo programa *Statistical Package for the Social Sciences* ^®^, versão 24, com licença adquirida pelos próprios autores do estudo.

## RESULTADOS

O estudo foi realizado com 198 técnicos de enfermagem, sendo a maioria do sexo feminino (95,5%) e com idade média de 37,2 anos. A maioria dos participantes tinha pele negra (81,8%), não tinha companheiro (60,6%) e tinha menos de 15 anos de educação formal (68,2%). Quanto à experiência profissional, 57,6% dos participantes tinham até seis anos de experiência. A maioria dos participantes relatou trabalhar até 12 horas por dia (66,7%). A maioria dos participantes (93,9%) classificou a qualidade do sono como má, e 43,9% relataram privação de sono, com menos de seis horas de sono. Em relação à memória, 37,9% dos participantes relataram falhas de memória geral, 10,6%, falhas de memória prospectiva, e 3%, falhas de memória retrospectiva. Ademais, as características da amostra são apresentadas na Tabela 1.

**Tabela 1:** Características sociodemográficas, laborais, comportamentais, clínicas, qualidade do sono e falhas de memória dos técnicos de enfermagem. Rio Verde, Goiás, Brasil, 2020-2021

Variáveis	N	%
Sexo		
Feminino	189	95,5
Masculino	9	4,5
Idade média (desvio padrão)	37,2	± 8,1
Cor da pele		
Branca	36	18,2
Negra	162	81,8
Estado civil		
Sem companheiro	78	39,4
Com companheiro	120	60,6
Escolaridade		
Menor de 15 anos	135	68,2
Maior de 15 anos	63	31,8
Tempo de profissão		
≤ seis anos	114	57,6
> seis anos	84	42,4
Horas/dia de trabalho		
≤ 12 horas	132	66,7
> 12 horas	66	33,3
Consumo de frutas, legumes e verduras		
Inadequado	81	40,9
Adequado	117	59,1
Consumo de alimentos ultraprocessados		
Não consome	63	31,8
Consome	135	68,2
Consumo de álcool		
Não	60	30,3
Sim	138	69,7
Consumo de café		
Não	33	16,7
Sim	165	83,3
Uso de tabaco		
Não	174	87,9
Sim	24	12,1
Índice de Massa Corporal		
Peso normal/baixo peso	78	39,4
Sobrepeso	63	31,8
Obesidade	57	28,8
Depressão		
Não	174	87,9
Sim	24	12,1
Ansiedade		
Não	114	57,5
Sim	84	42,4
Autopercepção de saúde		
Boa/muito boa	141	71,2
Ruim/péssima	57	28,8
Qualidade do sono		
Boa qualidade	12	6,1
Má qualidade	186	93,9
Privação do sono		
Sem privação/seis horas ou mais	111	56,1
Privação/menos de seis horas	87	43,9
Falhas de memória geral		
Ausente	123	62,1
Presente	75	37,9
Falhas de memória prospectiva		
Ausente	177	89,4
Presente	21	10,6
Falhas de memória retrospectiva		
Ausente	192	97
Presente	6	3

Ao realizar a avaliação da SDE na amostra de técnicos de enfermagem, constatou-se que 81 (40,9%, IC95%: 33,8 - 47,5) desses profissionais foram classificados com presença de SDE e 117 (59,7%, IC95%: 52,5 - 66,2) foram classificados com ausência de SDE.

Os resultados da regressão logística bivariada demonstram associações entre várias variáveis independentes com a SDE. Os profissionais com companheiro, com menor escolaridade, que consumiam alimentos ultraprocessados e álcool apresentaram maior probabilidade de ter SDE. O IMC também foi um fator relevante, com pessoas com sobrepeso e obesidade apresentando maior chance de ter SDE, em comparação com aquelas com peso normal ou baixo. O autorrelato de depressão, ansiedade, percepção ruim/péssima da saúde e falhas de memória geral também foi associado à SDE (Tabela 2).

**Tabela 2:** Frequência de sonolência diurna excessiva e associação com as variáveis sociodemográficas, laborais, comportamentais, clínicas, qualidade do sono e falhas de memória entre os técnicos de enfermagem. Rio Verde, Goiás, Brasil, 2020-2021

Variáveis	Presença de sonolência diurna excessiva n (%) 81 (40,9)	OR (IC95%)^*^	*p*
Sexo			
Feminino	75 (92,5)	0,32 (0,07 - 1,36)	0,124
Masculino	6 (7,4)	Referência	
Cor da pele			
Branca	15 (18,51)	Referência	
Negra	66 (81,48)	0,96 (0,46 - 2)	0,919
Estado civil			
Sem companheiro	24 (29,63)	Referência	
Com companheiro	57 (70,37)	2,03 (1,11 - 3,7)	0,020
Escolaridade			
Menor de 15 anos	63 (77,77)	2,18 (1,15 - 4,16)	0,017
Maior de 15 anos	18 (22,22)	Referência	
Tempo de profissão			
≤ seis anos	54 (66,66)	Referência	
> seis anos	27 (33,33)	0,52 (0,29 - 0,94)	0,032
Horas/dia de trabalho			
≤ 12 horas	51 (62,96)	Referência	
> 12 horas	30 (37,03)	1,32 (0,72 - 2,40)	0,358
Consumo de frutas, legumes e verduras			
Inadequado	36 (44,44)	1,28 (0,72 - 2,27)	0,400
Adequado	45 (55,55)	Referência	
Consumo de alimentos ultraprocessados			
Não consome	18 (22,22)	Referência	
Consome	63 (77,77)	2,18 (1,15 - 4,16)	0,017
Consumo de álcool			
Não	12 (14,81)	Referência	
Sim	69 (85,18)	4 (1,95 - 8,17)	<0,001
Consumo de café			
Não	15 (18,51)	Referência	
Sim	66 (81,48)	0,80 (0,37 - 1,70)	0,561
Uso de tabaco			
Não	69 (85,18)	Referência	
Sim	12 (14,81)	1,52 (0,64 - 3,58)	0,336
Índice de Massa Corporal			
Peso normal/baixo peso	21 (25,92)	Referência	
Sobrepeso	33 (40,74)	2,98 (1,47 - 6,03)	0,002
Obesidade	27 (33,33)	2,44 (1,18 - 5,02)	0,015
Depressão			
Não	63 (77,77)	Referência	
Sim	18 (22,22)	5,28 (1,99 - 14)	<0,001
Ansiedade			
Não	39 (48,14)	Referência	
Sim	42 (51,85)	1,92 (1,08 - 3,42)	0,026
Autopercepção de saúde			
Boa/muito boa	42 (51,85)	Referência	
Ruim/péssima	39 (48,14)	5,10 (2,62 - 9,93)	<0,001
Qualidade do sono			
Boa qualidade	3 (3,70)	Referência	
Má qualidade	78 (96,29)	2,16 (0,56 - 8,26)	0,258
Privação do sono			
Sem privação/seis horas ou mais	45 (55,55)	Referência	
Privação/menos de seis horas	36 (44,44)	1,03 (0,58 - 1,83)	0,905
Falhas de memória geral			
Ausente	33 (40,74)	Referência	
Presente	48 (59,26)	4,84 (2,61 - 8,99)	<0,001
Falhas de memória prospectiva			
Ausente	69 (85,18)	Referência	
Presente	14 (14,81)	2,08 (0,93 - 5,21)	0,115
Falhas de memória retrospectiva			
Ausente	75 (92,59)	Referência	
Presente	6 (7,40)	2,44 (0 - 0,01)	0,986

* *Odds Ratio (Intervalo de Confiança de 95%).*

Na análise de regressão logística multivariada, encontram-se na tabela as variáveis que mantiveram associadas com a SDE. Em relação ao sexo, as mulheres apresentaram uma chance 95% menor de ter SDE, em comparação com os homens (p = 0,001). Além disso, o tempo de profissão também foi um fator de proteção, no qual os profissionais com mais de seis anos de carreira tiveram 64% menor chance de ter SDE, em comparação com os com menor experiência (p = 0,020). O consumo de álcool mostrou uma associação significativa com a SDE, com uma chance 5,71 vezes maior para os consumidores de álcool em relação aos não consumidores (p = 0,002). O autorrelato de depressão também apresentou uma associação significativa com o desfecho, com uma chance de 3,66 vezes maior para aqueles com depressão, em comparação com aqueles sem depressão (p = 0,028). A autopercepção de saúde ruim/péssima mostrou uma associação significativa com o desfecho, com uma chance 15,41 vezes maior em comparação com aqueles que relataram uma percepção de saúde boa/muito boa (p < 0,001). Em relação à falha de memória geral, os indivíduos com falhas de memória tiveram uma chance 3,32 vezes maior de apresentar SDE, em comparação com aqueles que não apresentaram falhas de memória (p = 0,002) (Tabela 3).

**Tabela 3:** Associação de variáveis com a sonolência diurna excessiva entre os técnicos de enfermagem na análise de regressão logística multivariada. Rio Verde, Goiás, Brasil, 2020-2021

Variáveis	ORa (IC95%)^*^	*p*
Sexo		
Feminino	0,05 (0 - 0,29)	0,001
Masculino	Referência	
Tempo de profissão		
≤ seis anos	Referência	
> seis anos	0,36 (0,15 - 0,85)	0,020
Consumo de álcool		
Não	Referência	
Sim	5,71 (1,90 - 17,16)	0,002
Depressão		
Não	Referência	
Sim	3,66 (1,15 - 11,66)	0,028
Autopercepção de saúde		
Boa/muito boa	Referência	
Ruim/péssima	15,41 (5,37 - 44,27)	<0,001
Falhas de memória geral		
Ausente	Referência	
Presente	3,32 (1,52 - 7,25)	0,002

* *ORa = Odds Ratio ajustado (Intervalo de Confiança de 95%).*

## DISCUSSÃO

Os resultados deste estudo revelam uma prevalência elevada de SDE entre os técnicos de enfermagem juntamente com fatores sociodemográficos, laborais, comportamentais e clínicos associados a essa condição. Além disso, verificou-se uma associação significativa entre a SDE e falhas de memória geral, enquanto não foi observada uma associação com a qualidade do sono.

Dos técnicos de enfermagem avaliados, 81 (40,9%) apresentaram SDE. Essa prevalência é superior aos resultados encontrados em estudos realizados com a população geral, os quais também utilizaram o mesmo instrumento de avaliação. Esses estudos relataram uma prevalência de SDE variando entre 8,5% e 33%^(2,18)^. No entanto, em contexto brasileiro, pesquisa realizada com estudantes universitários da área da saúde identificou prevalência de 54,4% de SDE^(19)^, superior ao encontrado no presente estudo. Esses resultados demonstram a variabilidade de prevalência de SDE de acordo com a população avaliada, indicando a importância de avaliar especificamente a ocorrência desse problema entre técnicos de enfermagem.

Em relação aos profissionais de enfermagem, estudos realizados em diferentes cenários internacionais demonstraram prevalência de SDE de 25,6% entre enfermeiros da Noruega^(20)^, 28% entre enfermeiros da Inglaterra^(21)^ e 36,1% entre enfermeiros da Coréia do Sul^(22)^. Em estudos realizados com profissionais de enfermagem no Brasil, observou-se prevalência de SDE variando entre 30,09%^(23)^ e 38,6%^(9)^, valores inferiores ao encontrado no presente estudo. Entre as possíveis razões para a diferença nos resultados encontrados, podem referir-se ao momento em que foram realizadas as avaliações, tendo em vista que, no presente estudo, a coleta foi realizada durante a pandemia de covid-19. Não foram identificados estudos realizados em contexto nacional que avaliaram a SDE em profissionais da enfermagem durante a pandemia de covid-19. A pandemia de covid-19 influenciou a SDE entre os técnicos de enfermagem por diversos fatores, como o aumento do estresse psicológico, aumento da exposição ao vírus, carga de trabalho e alterações dos hábitos de sono e de vida^(22,24)^. Nesse sentido, há a necessidade do acompanhamento longitudinal desses profissionais com o objetivo de avaliar o impacto resultante da exposição aos fatores oriundos da pandemia para a saúde física e mental dos técnicos de enfermagem.

Além disso, é importante mencionar que a relação entre a SDE e a prática da enfermagem pode ser complexa e multifacetada. A natureza do trabalho de enfermagem, que muitas vezes envolve longas horas de trabalho, turnos noturnos, ritmo acelerado e demandas físicas e emocionais intensas, pode contribuir para a ocorrência da SDE^(25)^.

Embora não tenha sido observada associação significativa com a SDE, é importante ressaltar que a maioria expressiva da amostra de técnicos de enfermagem apresentou má qualidade do sono, representando um total de 186 indivíduos (93,9%). Esses resultados indicam que a população estudada está suscetível a problemas relacionados à qualidade do sono^(26)^, mesmo que não estejam diretamente associados à ocorrência de SDE. Pesquisa recente envolvendo 42 enfermeiros hospitalares no sul do Brasil revelou que 64,3% dos profissionais apresentaram má qualidade do sono^(27)^. Além disso, outro estudo conduzido com profissionais de enfermagem identificou uma prevalência de 75% dos indivíduos avaliados com distúrbio do sono^(25)^. Esses achados corroboram os resultados encontrados no presente estudo, destacando a preocupante situação da qualidade do sono entre os profissionais de enfermagem no cenário nacional. A alta proporção de profissionais de enfermagem com má qualidade do sono pode ser um reflexo das condições laborais desafiadoras e do impacto da rotina de trabalho intensa e irregular sobre o descanso adequado^(28-29)^.

Entre as covariáveis que apresentaram associação significativa com a SDE, as mulheres e os profissionais com maior tempo de experiência tiveram menores chances de apresentarem tal problemática. Mulheres possuem menor probabilidade de ter SDE devido a padrões regulares e melhor qualidade do sono em relação aos homens^(30)^. Além disso, a experiência profissional pode estar associada à SDE, possivelmente devido ao desenvolvimento de estratégias de gerenciamento do sono ao longo dos anos, mitigando os impactos negativos^(31)^.

As outras covariáveis associadas à SDE entre os técnicos de enfermagem foram o consumo de álcool, o autorrelato de depressão e a autopercepção negativa da saúde. Em relação ao álcool, apesar de possuir propriedades sedativas e hipnóticas, seu consumo pode levar à fragmentação, alteração e redução da qualidade do sono, o que aumenta as chances de desenvolvimento de SDE. Há evidências que o consumo de álcool pode interferir nos processos do sono, afetando negativamente a sua estrutura e duração^(32)^. No contexto da depressão, os resultados estão em concordância com os dados encontrados em estudo realizado na China com enfermeiros de um hospital geral, onde demonstrou a depressão como um preditor da ocorrência de SDE^(11)^. É importante mencionar que a SDE e a depressão podem ter uma relação bidirecional, no qual cada uma influência o outro, sendo esse fato constatado em estudos que apontam os efeitos dos problemas do sono na depressão e vice-versa^(33)^. Ademais, a associação da autopercepção negativa da saúde com a SDE pode ser indicativa das consequências da má qualidade do sono e do alto índice de SDE, fatores que resultam em déficits cognitivo e alterações de humor, que podem levar uma avaliação mais negativa da saúde.

Os resultados do presente estudo demonstram associação significativa entre SDE e falhas de memória geral, mesmo após o controle de possíveis fatores de confusão. No entanto, não foram encontradas associações entre a SDE e a memória prospectiva e retrospectiva. A associação encontrada está em consonância com a literatura científica sobre sono, que tem examinado extensivamente a relação entre o sono e a eficiência das funções executivas, incluindo o desempenho da memória. Revisão sistemática recente destacou a influência dos aspectos do sono no funcionamento da memória, reforçando ainda mais a importância dessas descobertas^(8)^. Corroborando os resultados da presente pesquisa, estudo realizado com idosos no Japão evidenciou a SDE como um importante preditor de comprometimento subjetivo da memória^(34)^.

Uma possível explicação para a associação entre SDE e falhas de memória está relacionada aos déficits de atenção e concentração decorrentes da SDE. Indivíduos que apresentam SDE tendem a ter dificuldades em manter o foco e sustentar a atenção ao longo do dia, o que pode afetar negativamente o desempenho da memória^(1)^. Quando estão excessivamente sonolentos durante o dia, eles podem encontrar dificuldades na codificação adequada das informações, bem como na recuperação efetiva das mesmas durante as tarefas de memória. Esses déficits de atenção e concentração podem resultar em falhas de aquisição, retenção e recuperação das informações armazenadas na memória, comprometendo, assim, o funcionamento cognitivo relacionado à memória^(35)^.

É importante mencionar que a SDE associada com falhas de memória, entre os técnicos de enfermagem, ocasiona impactos significativos na qualidade de vida, no desempenho profissional e na segurança dos pacientes. Essa problemática pode afetar a capacidade de concentração, a tomada de decisões, a coordenação motora e a vigilância, o que pode levar a erros e acidentes no ambiente de trabalho^(11)^. Os resultados de estudo realizado com enfermeiros de uma unidade hospitalar da China demonstraram que a SDE estava associada ao aumento de 1,83 vezes da ocorrência de eventos adversos^(11)^. Esses achados sustentam a teoria de que a SDE entre os profissionais de enfermagem representa um risco significativo para a segurança do paciente, devido ao aumento da probabilidade de eventos adversos durante a prática profissional^(36)^.

Em razão dos elevados índices de SDE evidenciados no presente estudo, cabe ressaltar a necessidade de avaliação contínua dos aspectos relacionados ao sono entre os técnicos de enfermagem no período pós-pandêmico. Sendo assim, é imprescindível reconhecer que a problemática relacionada à SDE pode persistir ou evoluir em eventos subsequentes, exigindo atenção especial e estratégias preventivas para mitigar seus impactos na saúde, no sono e no desempenho profissional dos técnicos de enfermagem.

### Limitações do estudo

O presente estudo deve ser interpretado considerando algumas limitações relevantes que podem impactar os resultados e sua generalização. A amostra utilizada neste estudo foi restrita a uma única instituição de saúde, o que pode limitar a representatividade dos resultados. A utilização de questionários de autorrelato pode resultar em um viés de memória e que pode influenciar a precisão das associações observadas. Outras limitações do estudo se referem ao fato de não ter sido realizada uma avaliação detalhada com instrumento validado sobre o consumo de álcool e, também, relacionada à presença onipresente de viés, uma vez que os processos cognitivos, a desejabilidade social e as condições específicas podem influenciar as respostas dos participantes.

Dessa forma, são necessários estudos futuros que considerem essas limitações apresentadas. Novas pesquisas com amostras mais representativas, delineamentos longitudinais e o uso de instrumentos mais robustos para avaliar a qualidade do sono, a SDE e a memória são necessários para fornecer evidências mais robustas e esclarecer a relação entre essas variáveis.

### Contribuições para as áreas da enfermagem, saúde ou política pública

Os resultados da presente pesquisa possuem importantes implicações práticas. A alta prevalência de SDE e a associação com falhas de memória destacam a necessidade das instituições e gerentes de saúde de fornecerem educação e conscientização aos profissionais de enfermagem sobre os riscos associados ao SDE tanto para a saúde do profissional quanto para a saúde dos pacientes assistidos. É essencial incluir treinamentos que visem à elaboração de estratégias para melhorar a qualidade do sono entre os técnicos de enfermagem. Uma abordagem promissora nesse sentido é a prática de atenção plena (*mindfulness*), que consiste em exercícios de baixa densidade que podem ser facilmente incorporados na rotina desses profissionais^(37-38)^. Estudos têm demonstrado que a prática regular de *mindfulness* pode melhorar a qualidade do sono, reduzir a sonolência diurna e promover o bem-estar geral. As instituições de saúde devem realizar ações de rastreamento e avaliação da SDE entre os profissionais de enfermagem e fornecer suporte e promover uma cultura de segurança para incentivarem os profissionais a relatarem os problemas relacionados à SDE por meio de uma comunicação aberta e apoio mútuo.

Os achados do estudo, nos quais identificaram 40,9% de SDE e associação com falhas de memória entre técnicos de enfermagem, demonstram que a SDE é um problema de saúde pública que pode afetar o desempenho profissional e a qualidade de vida dos profissionais de saúde. Esses resultados sugerem a necessidade de proporcionar suporte para a categoria de profissionais de saúde e, principalmente, enfermagem, especialmente em períodos pós-pandêmicos. Esse suporte deve abranger intervenções psicossociais, além de programas de bem-estar e estratégias de mitigação de potenciais impactos da prática profissional para a saúde mental desses profissionais. Além disso, recomenda-se que futuras pesquisas avaliem aspectos detalhados relacionados à prática profissional, explorando intervenções psicossociais visando à diminuição da SDE e que utilizem abordagens eficazes visando às falhas de memória associadas à SDE.

## CONCLUSÕES

Os resultados do presente estudo demonstram a elevada ocorrência de SDE e a associação com falhas de memória geral entre os técnicos de enfermagem em modelos multivariados.

A alta proporção de técnicos de enfermagem com SDE e a associação com falhas de memória reforçam a necessidade de intervenções e políticas de saúde voltadas para a promoção de um sono adequado e saudável nessa população, visando melhorar sua qualidade de vida e desempenho no ambiente de trabalho.
